# Supplementary material for: Dietary practice and associated factors among elderly people in Northwest Ethiopia, 2022: Community based mixed design
Source: PLoS One. 2024 Aug 27;19(8):e0307748. doi: 10.1371/journal.pone.0307748 (PMC11349100; doi:10.1371/journal.pone.0307748)
Supplement: Attachment — Submitted filename: comments.pdf [file pone.0307748.s002.pdf]

General comment:

The article is well structured, with a clear rationale, robust methodology and comprehensive analysis. It makes a valuable contribution to understanding the dietary practices of older people in northwestern Ethiopia. However, future research could benefit from longitudinal designs, validation of data collection instruments, and exploration of additional regions for wider applicability.

Specific comments:

Methods:

- Data Collection: There is no information in the document as to whether the tools have been validated or whether there have been any pre-testing procedures.

Results:

-It is recommended that the clarity of tables and figures be enhanced and that qualitative findings are presented in more detail in the abstract and discussion.

Discussion:

- Potential limitations of this study include the possibility of self-reported data biases, the cross-sectional nature of the study (which may limit causal inferences), and the possibility of non-generalizability to other regions.

- It is recommended that the findings be integrated in the discussion in order to provide a more holistic view. This integration should include both qualitative and quantitative findings.
