# Supplementary material for: Dietary practice and associated factors among elderly people in Northwest Ethiopia, 2022: Community based mixed design
Source: PLoS One. 2024 Aug 27;19(8):e0307748. doi: 10.1371/journal.pone.0307748 (PMC11349100; doi:10.1371/journal.pone.0307748)
Supplement: Attachment — Submitted filename: Rebuttal letter.docx [file pone.0307748.s001.docx]

**Rebuttal letter**

**Title:** **DIETARY PRACTICE AND ASSOCIATED FACTORS AMONG ELDERLY PEOPLE IN NORTHWEST ETHIOPIA, 2022: COMMUNITY BASED MIXED DESIGN.**

**Ms. Ref. No.:** PONE-D-23-36778

We like to thank the reviewer for the positive and constructive comments with regard to the submission for publication of our manuscript in PLOS ONE**.** We have tried to address all comments and suggestions of the reviewer’s point by point by **writing question number** and **answer** for each and revised our manuscript accordingly. Revisions to the text were highlighted in the manuscript.

**Reviewer #1**

1. In the background-introduction, I recommend to amplify the references number. You mentioned some importants aspects in diet policy.

**Answer**: Thank you very much for your valuable comments. We have addressed your concerns and made correction.

2. I think that you must talk about lifespan in Ethiopia, and also the main differences between develop and undeveloped countried in the food frange

**Answer:** Thank you very much for your valuable comments. We have addressed your concerns and made correction.

3. You have to comment also main food and lifestyle characteristics of the study population, because only with food profile we could not understand well some differences between religion, occupation, etc.

**Answer**: Thank you very much for your valuable comments. We have addressed your concerns and made correction.
4. Maybe, I suggest that they could explain better why they did qualitative part in only 26 person and what factors or characters are relevant in this 26 person. Maybe results could be different in the way of this qualitative test.
**Answer:** Thank you very much for your valuable comments.

We have addressed your concerns in the method and material section. In determining the sample size of the qualitative research, what matters was theoretical data saturation. In our case the idea reached saturation on 26^th^ individuals.

**5.** Also, I suggest to add an image about FCS.

**Answer:** Thank you very much. We have addressed your suggestions.
