# Supplementary material for: Dietary practice and associated factors among elderly people in Northwest Ethiopia, 2022: Community based mixed design
Source: PLoS One. 2024 Aug 27;19(8):e0307748. doi: 10.1371/journal.pone.0307748 (PMC11349100; doi:10.1371/journal.pone.0307748)
Supplement: Attachment — Submitted filename: Rebuttal letter_1.docx [file pone.0307748.s003.docx]

**Rebuttal letter**

**Title:** **DIETARY PRACTICE AND ASSOCIATED FACTORS AMONG ELDERLY PEOPLE IN NORTHWEST ETHIOPIA, 2022: COMMUNITY BASED MIXED DESIGN.**

**Ms. Ref. No.:** PONE-D-23-36778R1

We like to thank the academic editor and reviewers for the positive and constructive comments with regard to the submission for publication of our manuscript in PLOS ONE**.** We have tried to address all comments and suggestions of the academic editor’s and reviewers’ point by point by **writing question number** and **answer** for each and revised our manuscript accordingly. Revisions to the text were highlighted in the manuscript.

**Academic Editor**

1. General comment: strictly adhere with the journals submission gudline. Give continuous line numbers to easily locate comments. For example, the fourth paragraph of the first sentence, written as "In the global food system," is an incomplete sentence or inappropriate punctuation.

**Response:** thank very much. We amended it accordingly.

2. Abstract: Include the 95% CI for the prevalence of adequate dietary practice.

**Response:** Thank you. We included it.

3. Method: the sampling procedure is unclear. What was your primary sampling unit? households or individual elderly? What was the total number of households in the town? Of which, what was the total number of households with at least one elderly person? As you know, in Ethiopian culture, when the grandparents are old enough, they jointly live in their children's house ("tibatie") with a monthly or more round for each available child. How did you address them? Even, how the K value is applied is unclear? is it for the household or for each individual? If so, the K value is 4.58, which is rounded to 4, but you selected every two. How did you select, if both couples were elderly?

**Response:** Thank you very much. We amended it. It was a typography error for the K interval calculation. As you calculated the K value is 4.58.

The sampling interval of households in each kebele was determined by dividing the total number of households which has eligible elderly people to the allocated sample size meaning to select the study units (elderly people). Systematic random sampling was used with sampling interval k^th^ which was calculated as k = 1933/422 = 4.58 = 5.

Out of the first 5 elderly people, the initial one was determine by using the lottery method. For households with multiple elderly people, one elderly person was selected by lottery method.

Finally, a systematic random sampling technique was used for the selection of individual study participants until having the sample of 422 participants.

We included these statements in the main manuscript.

4. Result: Modify the interpretations; do not interpret OR as RR. For example, "Elders aged 65 to 74 years were 8.32 times more likely to have adequate dietary practice (AOR:8.32; 95 CI: 3.9, 18.1) than elders aged greater than or equal to 85 years". It would be better if you interpreted the odds of adequate dietary practice as 8.32 times (AOR:8.32; 95 CI: 3.9, 18.1) higher among elders aged 65 to 74 years compared to 85 years and above.

Table 4: Locate the reference categories with a value of 1 rather than making the cells empty.

**Response:** Thank you very much for the comment. We amended it accordingly.

**Reviewer #2**

1. Why one admstrative town considered and why not didn't considered the rural area of the district. This need critical justification.

**Answer**: Thank you very much for your valuable comments. It was basically due to resource limitation. Since there is no study conducted in the area the findings used for baseline to further research in wide area. The administrative town have dense population than rural this have a good chance to have a representative sample with a small budget. Not only this but accessibility often better in town than rural areas.

2. The result should be discussed with similar study conducted elsewhere and looks like shallow.

**Response:** thank you very much we considered your valuable comments.

3. Make sure that no study conducted in Ethiopia and you took 50% during sample size calculation

**Response:** Thank you very much for your valuable comments. We have addressed your concerns. As to our effort we could not found similar study in Ethiopia.

**Reviewer #3**

1. Methods: - Data Collection: There is no information in the document as to whether the tools have been validated or whether there have been any pre-testing procedures.

**Response:** Thank you very much**.** We included your comments. It was pre- tested other than of the study area with 5% of the sample size.

2. Results: -It is recommended that the clarity of tables and figures be enhanced and that qualitative findings are presented in more detail in the abstract and discussion.

**Response:** Thank you very much. We considered your comments and amend it accordingly.

3. Discussion: - Potential limitations of this study include the possibility of self-reported data biases, the cross-sectional nature of the study (which may limit causal inferences), and the possibility of non-generalizability to other regions.

- It is recommended that the findings be integrated in the discussion in order to provide a more holistic view. This integration should include both qualitative and quantitative findings.

**Response:** Thank you very much. We considered your comments and amend it accordingly.
