# Supplementary material for: Dietary practice and associated factors among elderly people in Northwest Ethiopia, 2022: Community based mixed design
Source: PLoS One. 2024 Aug 27;19(8):e0307748. doi: 10.1371/journal.pone.0307748 (PMC11349100; doi:10.1371/journal.pone.0307748)
Supplement: Attachment — Submitted filename: Rebuttal letter_last.docx [file pone.0307748.s004.docx]

**Rebuttal letter**

**Title:** **DIETARY PRACTICE AND ASSOCIATED FACTORS AMONG ELDERLY PEOPLE IN NORTHWEST ETHIOPIA, 2022: COMMUNITY BASED MIXED DESIGN.**

**Ms. Ref. No.:** PONE-D-23-36778R1

We like to thank the academic editor and reviewers for the positive and constructive comments with regard to the submission for publication of our manuscript in PLOS ONE**.** We have tried to address all comments and suggestions of the academic editor’s and reviewers’ point by point by **writing question number** and **answer** for each and revised our manuscript accordingly. Revisions to the text were highlighted in the manuscript.

**Academic Editor**

1. Please review your reference list to ensure that it is complete and correct. If you have cited papers that have been retracted, please include the rationale for doing so in the manuscript text, or remove these references and replace them with relevant current references. Any changes to the reference list should be mentioned in the rebuttal letter that accompanies your revised manuscript. If you need to cite a retracted article, indicate the article’s retracted status in the References list and also include a citation and full reference for the retraction notice.

**Response:** Thank very much. We amended it accordingly.

2. You addressed all the comments raised in the previous round of revisions. Your manuscript needs one last proofread. In the previous revision, I suggested some modifications to the interpretation, and you did it well. However, it still needs some modifications. Eg., line 269, "The odds of adequate dietary practice as 8.32 times (AOR: 8.32; 95 CI: 3.9, 18.1) higher among elders aged 65 to 74 years compare 85 years and above. Actually, it was my mistake too. So, please modify as: The odds of adequate dietary practice were 8.32 times (AOR: 8.32; 95 CI: 3.9, 18.1) higher among elders aged 65 to 74 years, compared to 85 years and above. Modify all the rest with the same or better approach.

**Response:** Thank you. We modify it.
